# miRNA-Based Therapeutics in Breast Cancer: A Systematic Review

**DOI:** 10.3389/fonc.2021.668464

**Published:** 2021-05-05

**Authors:** Anna Maria Grimaldi, Marco Salvatore, Mariarosaria Incoronato

**Affiliations:** IRCCS SDN, Naples, Italy

**Keywords:** microRNA, breast cancer, miRs cancer therapy, miR-mimics, antagomirs, diagnosis, miRNA target, animal model

## Abstract

**Background:**

Breast cancer (BC) is the most common cancer in females and despite advances in treatment, it represents the leading cause of cancer mortality in women worldwide. Conventional therapeutic modalities have significantly improved the management of BC patients, but subtype heterogeneity, drug resistance, and tumor relapse remain the major factors to hamper the effectiveness of therapy for BC. In this scenario, miRNA(miR)-based therapeutics offer a very attractive area of study. However, the use of miR-based therapeutics for BC treatment still represents an underdeveloped topic. Therefore, this systematic review aims at summarizing current knowledge on promising miR-based therapeutics for BC exploring original articles focusing on *in vivo* experiments.

**Methods:**

The current systematic review was performed according to PRISMA guidelines. PubMed and EMBASE databases were comprehensively explored to perform the article search.

**Results:**

Twenty-one eligible studies were included and analyzed: twelve focused on antitumor miR-based therapeutics and nine on metastatic miR-based therapeutics. We found 18 different miRs tested as potential therapeutic molecules in animal model experiments. About 90% of the selected studies evaluate the efficiency and the safety of miRs as therapeutic agents in triple-negative (TN)-BC mouse models. Among all founded miR-based therapeutics, miR-21 emerged to be the most investigated and proposed as a potential antitumoral molecule for TNBC treatment. Besides, miR-34a and miR-205a appeared to be successful antitumoral and antimetastatic molecules.

**Conclusions:**

Our analysis provides a snapshot of the current scenario regarding the miRs as therapeutic molecules in BC. Nevertheless, despite many efforts, none of the selected studies goes beyond preclinical studies, and their translatability in the clinical practice seems quite premature.

## Introduction

Breast cancer (BC) is a heterogeneous and complex disease, ranked the second most common cancer worldwide, with an estimated 2,261,419 new cases and 684,996deaths annually (1). The conventional protocols for BC treatment include local therapies such as surgery, radiation, and systemic treatments like chemotherapy, endocrine therapy, targeted therapy, and more recent immunotherapy (2). Although these therapeutic modalities have significantly improved the survival of BC patients, their responses may be heterogeneous, as some patients benefiting whereas others respond less. It is clinically established that different subtypes may respond differently to therapies (3). For instance, treatment options for BC triple-negative subtype (TNBC) are limited: hormone therapy and drugs that target HER2 are not helpful, and chemotherapy is the main systemic treatment option. Although TNBC seems to respond well to initial chemotherapy, it tends to recur more frequently than other BC subtypes. Indeed it is associated with a high risk of recurrence and distant metastases to the brain, bone and lung with poor outcomes compared to other BC subtypes (4).

Nonetheless, in addition to subtypes heterogeneity limit, drug resistance and tumor relapse remain the major factors that hamper the disease-free survival of BC patients, indicating the need for new alternative approaches for treating BC. Among the plethora of attractive tools and targets for novel therapeutic approaches, miRNAs (miRs) seem to hold great potential in anticancer therapy. MiRs are members of a large class of non-coding RNAs of approximately 19 to 24 nucleotides in length that regulate gene expression at the post-transcriptional level (5). A single miR can target and regulate up to hundreds of genes, each of which could be involved in biological pathways pathogenically disrupted in a diseased patient. Their pleiotropic role as well as their dysregulation in pathological conditions, provides further support to miRs as future therapeutics especially for diseases that are not caused by a single genetic link (6–8). miR therapeutics are oligonucleotides that modulate the function of miRs, correcting the imbalance of gene expression and associated cellular pathways (9). In the last decades, many preclinical studies have suggested that the therapeutic modulation of miRs, achieved by the inhibition of oncogenic miRs and the replacement of deficient tumor-suppressive miRs, could represent a reliable tool for improving cancer treatment. To date, candidate miR therapeutics are in clinical development or phase I and phase II clinical trials. Nevertheless, they have not yet translated into FDA-approved candidates for medical intervention and the use of miR-based therapeutics for BC treatment still represents an underdeveloped topic. Thanks to the growing collection of animal models, the preclinical validation of miRs for therapeutic purposes becomes more and more robust, and strong findings could represent the premise for new clinical trials. So, here we provide a systematic overview to collect and summarize the current state of the art on miR-based therapeutics in BC animal models, for assessing the miR skills to be antitumoral and antimetastatic drugs.

## Materials and Methods

### Search Strategy

The current systematic review was performed according to the guidelines of the Preferred Reporting Items for Systematic Reviews and Meta-Analyses (PRISMA) statement (10) (see **Supplementary Table 2** for PRISMA Checklist). A systematic search for all published studies concerning miR-based therapeutic approaches for BC *in vivo* was conducted independently from two authors (AMG and MI). Two scientific electronic databases, PubMed and EMBASE, were comprehensively explored to perform the articles search. The search strategy complete of key terms is listed in **Supplementary Table 1**. Besides, for the identification of any relevant and eligible articles, we performed a manual search from the bibliography of all included articles.

### Selection Criteria

Two researchers (AMG and MI) independently, after having screened identified studies for titles and abstracts, included or excluded articles basing on the following inclusion/exclusion criteria. Inclusion criteria: (1) therapeutic approach of miR in BC; (2) original articles; (3) English language. Exclusion criteria: (1) letters, case reports, reviews, conference abstracts; (2) non-English papers; (3) studies performed only on immortalized BC cell lines; (4) studies not focused on BC; (5) methodological studies. On failure to reach an agreement between the reviewers, a third reviewer (MS) was consulted for advice.

### Data Extraction and Collection

After the selection procedure of papers, data that met the inclusion criteria were summarized into a customized Excel spreadsheet database by two investigators independently (AMG and MI) and then compared to each other. For each study, the following characteristics were collected: miR-based treatment (miR-replacement therapy and/or miR-inhibition therapy); miR target; methodological approach; *in vitro* BC model tested; gene(s)/pathway regulated; *in vivo* BC model tested; delivery system; biological effect and reference.

## Results

### Literature Search Results

**Figure 1:**
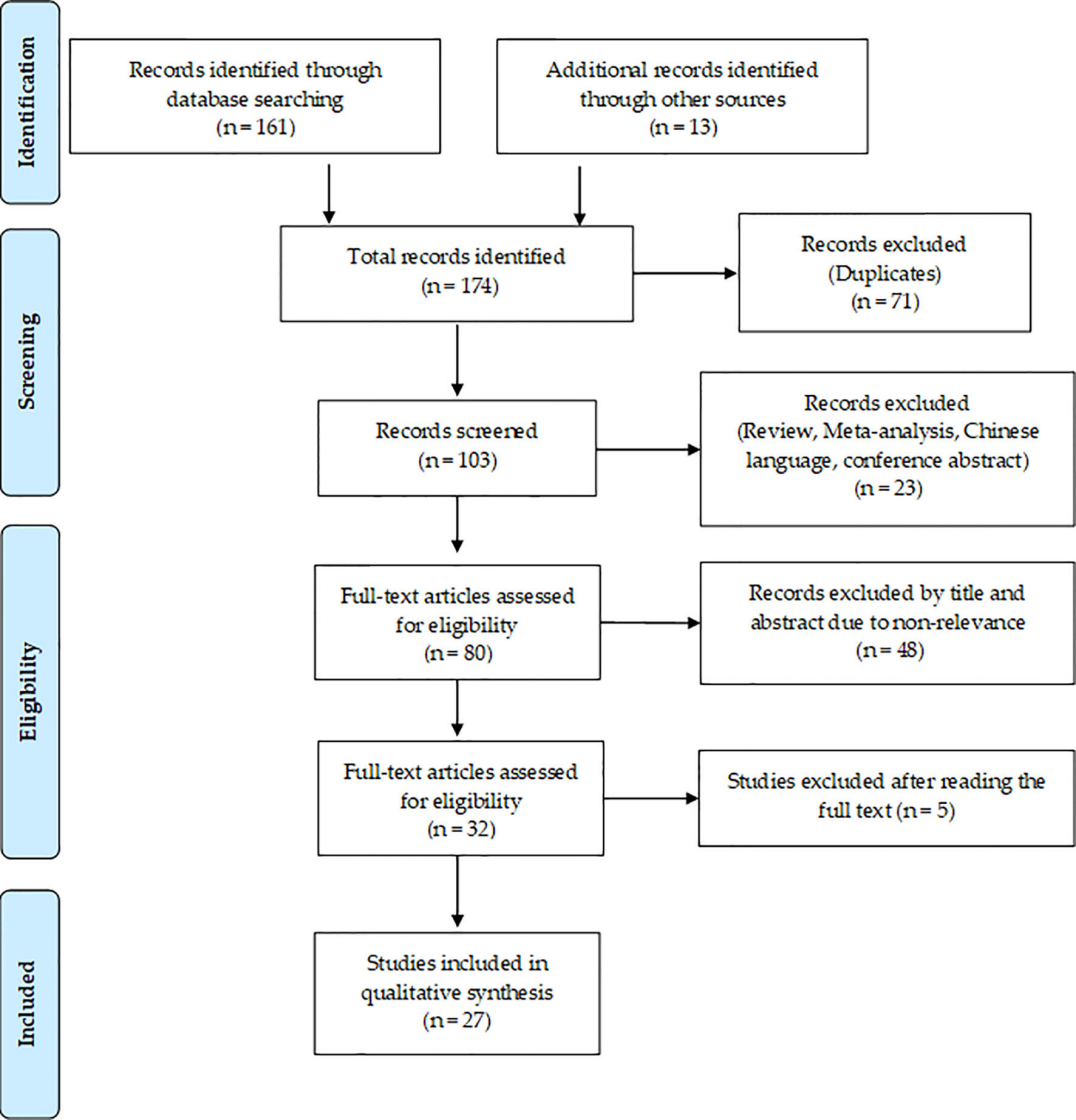
Flowchart for the strategy searches and selection processes.

A flowchart showing the publication search and the detailed selection process of the articles is reported in **Figure 1**. The oldest studies provided by both queried databases dated back to 2010. A total of 174 potential eligible records related to the miR-based therapeutic approaches for BC were retrieved from PubMed and EMBASE public databases and additional sources in the initial search, such as relevant studies identified by references of other scientific papers. Then, 71 duplicates were deleted, of the remaining 103 records, 23 of them were excluded as non-research articles or non-English literature publications. From the remaining 80 articles, 48 were excluded because they were irrelevant after screening the title and abstract. The remaining 27 eligible articles were downloaded and read, and five of them were excluded due to the paucity of sufficient information. The final 27 eligible articles were grouped according to two fields of study: 1) antitumor miR-based therapeutics and 2) antimetastatic miR-based therapeutics. Overall, 19 miRs were identified for therapeutic applications. Specifically, 12 miRs (miR-497, miR-544, miR-34a, let-7b, miR-603, miR-203, miR-26a, miR-142-3p, miR-21, miR-221 and miR-205) were accepted as potential antitumor miR-based therapeutics (**Table 1**), 7 miRs (miR-214, miR-222, miR-223, miR-205-5p, miR-100, miR-4306, miR-708 and miR-3613-3p) as potential antimetastatic miR-based therapeutics (**Table 2**) and 2 (miR-10b and miR-34a) as molecules potentially able to have both antitumor and antimetastatic role in BC treatment (**Tables 1** and **2**).

**Table 1:** miR-based therapy and their role as anticancer drugs in BC treatment.

miR-based treatment	miR target	Methodological approach	*In vitro* BC model tested	Gene(s)/pathway regulated	*In vivo* BC model tested	Delivery system	Biological effect	Ref.
miR-replacement therapy	miR-497	miR-497 mimic	T74D, MCF-7, MDA-MB-453, MDA-MB-468, MDA-MB-435, MCF10A	VEGF, HIF-1α	Xenograft mouse (MCF-7)	Transfection and subcutaneously injection	Inhibited tumor growth and suppressed angiogenesis	Wu et al. (11)
	miR-544	miR-544 mimic	HCC38, HCC1143, HCC1395, MCF-10A, HMEC	BCL6, STAT3	Xenograft mouse (HCC38, HCC1143, HCC1395)	Transfection and subcutaneously injection	Inhibited tumor growth	Zhu et al. (12)
	miR-34a	T-VISA-miR-34a	MDA-MB-231, MDA-MB-361, MDA-MB-435, MDA-MB-468, BT-20, BT-474, BT-483, MCF-7, HBL-100, AU565, SK-Br3, T47D, ZR-75-1, 184A1, MCF-10A	E2F3, CD44, SIRT1	Orthotopic xenograft mouse (MDA-MB-231)	Liposomal NPs and intravenously injection	Inhibited tumor growth and prolonged survival	Li et al. (13)
	miR-34a	miR-34 mimic	CRC-Normal, MCF-7, BT-474, MDA-MB-453, MDA-MB-231, MDA-MB-157, MDA-MB-436, Hs578T, BT-549, BT-20	c-SRC signaling pathway	Orthotopic xenograft mouse (MDA-MB-231)	Neutral-lipid emulsion intratumorally injection	Inhibited tumor proliferation and invasion, activated senescence, and sensitized mesenchymal-TNBC cells to dasatinib	Adams et al. (14)
	miR-34a	miR-34 mimic	MDA-MB-231	Bcl2, Notch1	Xenograft mouse (MDA-MB-231)	Hyaluronic acid-chitosan NPs subcutaneously injection	Inhibited tumor growth and suppression of cells migration	Deng et al. (15)
	let-7b	miR-let-7b mimic	–	TLR-7, IL-10	Orthotopic mouse (4T1)	PHA NPs and intravenously injection	Inhibited tumor growth and reversed the suppressive tumor microenvironment	Huang et al. (16)
	miR-603	miR-603 mimic	MDA-MB-436, MDA-MB-231, MDA-MB-468, BT-549, BT-20, MCF-10A	eEF2K	Orthotopic xenograft mouse (MDA-MB-436)	Peg-liposomal NPs and intravenously injection	Reduced tumor growth	Bayraktar et al. (17)
	miR-203	miR-203 mimic	MDA-MB-231	SLUG, TGF-β1/SMAD pathway	Xenograft mouse (MDA-MB-231)	Liposomal NPs and intravenously injection	Induced apoptosis and combined therapy with vinorelbine resulted in a nearly complete anticancer effect	Yan et al. (18)
	miR-26a	miR-26a mimic	MDA-MB-231	BAK1	Xenograft mouse (MDA-MB-231)	Aptamer-chimera and intravenously injection	Repressed tumor growth and synergized with 5-FU or carboplatin in cancer therapy protecting the host against myelosuppression by chemotherapy	Tanno et al. 19
miR-inhibition therapy	miR-142-3p	LNA-based antagomiR-142-3p	TUBO	APC, P2X7R	Orthotopic mouse (4T1)	Exosomes and intravenously injection	Inhibited tumor growth	Naseri et al. 20
	miR-21	antagomiR-21	4T1	PTEN, HIF-1α/VEGF/VEGFR2 pathway	Orthotopic mouse (4T1)	Transfection and intravenously injection	Inhibited angiogenesis and tumor growth	Zhao et al. (21)
	miR-21	LNA-based antagomiR-21	MDA-MB-231	PTEN, PDCD4	Orthotopic xenograft mouse (MDA-MB-231)	Multifunctional RNA NPs and intravenously injection	Inhibited tumor growth	Yin et al. (22)
	miR-21	antagomiR-21	MDA-MB-231, MCF-7, MCF-10A, MDA-MB-231 CSCs	Nanog, Oct4, Sox2	Xenograft mouse (MDA-MB-231)	PAMAM modified gold NPs and intravenously injection	Inhibited tumor growth	Ren et al. (23)
	miR-21	antagomiR-21	MCF-7	–	Xenograft mouse (MCF-7)	Polymeric NPs and intravenously injection	Inhibited tumor growth	Bahreyni et al. (24)
	miR-10b, miR-21	antisense-miR-21 and antisense-miR-10b	MDA-MB-231	–	Xenograft mouse (MDA-MB-231)	Polymeric NPs and intravenously injection	Reduced tumor growth	Devulapally et al. (25)
miR-inhibition/miR-replacement therapy	miR-221, miR-205	antagomiR-221 and miR-205 mimic	MDA-MB-231	–	Orthotopic xenograft mouse (MDA-MB-231)	RNA-triple-helix hydrogel scaffold and subcutaneously implanted	Reduced tumor growth	Conde et al. (26)

**Table 2:** miR-based therapy and their role as antimetastatic drugs in BC treatment.

miR-based treatment	miR target	Methodological approach	*In vitro* BC model tested	Gene(s)/pathway regulated	*In vivo* BC model tested	Delivery system(administration route)	Biological effect	Ref.
miR-inhibition therapy	miR-10b	antagomiR-10b	4T1	–	Orthotopic mouse (4T1)	Transfection and intravenously injection	Suppressed formation of lung cancer metastasis	Ma et al. (27)
	miR-10b	LNA-based antagomiR-10b	MDA-231-BoM-1833, MDA-231-LM2-4175, MDA-231-BrM2-831, MDA-MB-231-luc-D3H2LN	HOXD10	Orthotopic mouse (4T1)	Iron oxide NPs and intravenously injection	reduced pre-existing distant metastases and cancer mortality	Yoo et al. (28)
	miR-10b	antagomiR-10b	4T1	HOXD10	Orthotopic mouse (4T1)	Liposomes (PEG-Lip) intravenously injection	Delayed the growth of the primary tumor and reduced the lung metastases	Zhang et al. (29)
	miR-214	antagomiR-214 (R97 or R98)	4175-TGL	–	Orthotopic mouse (4175-TGL)	Unassisted uptake (chemically modified miR) and intravenously injection	Reduced number of circulating tumor cells and lung or lymph node metastasis formation	Dettori et al. (30)
	miR-222, miR-223	antagomiR-222/223	MDA-MB-231, T47D	–	Xenograft mouse (MDA-MB-231 or T47D)	Transfection and intravenously injection	sensitized BC to carboplatin-based therapy and increased survival	Bliss et al. (31)
	miR-205-5p	LNA-based antagomiR-205-5p	cell from tissue patients	–	Xenograft mouse (BC stem cells)	Unassisted uptake (chemically modified miR) and intraperitoneally injection	Impaired tumor growth and reduced the number and the size of lung metastasis	De Cola et al. (32)
	miR-100	antagomiR-100	4T1, RAW264.7	mTOR/Stat5a pathway	Orthotopic mouse (4T1)	Unassisted uptake (chemically modified miR) and intratumorally injection	Inhibited lung metastasis and enhanced chemotherapy-sensitivity	Wang et al. (33)
miR-replacement therapy	miR-4306	miR-4306 mimic	ZR-75-1, MCF-7, T47D, SK-BR-3, HCC1937, MDA-MB-468, MDA-MB-231, CAL-51	SIX1, Cdc42, VEGFA	Orthotropic xenograft mouse (CAL-51)	Cholesterol-conjugated and intratumorally injection	Inhibited TNBC cell growth, lung metastasis, angiogenesis and lymphatic metastasis	Zhao et al. (34)
	miR-708	miR-708 mimic	293, 4T1 and MDA-MB-231	–	Orthotopic mouse (MDA-MB-231-LM2)	Multilayer AuNPs and intratumorally injection	reduced lung metastasis formation	Ramchandani et al. (35)
	miR-34a	tRNA-miR-34a	4T1.2, MDA-MB-231	–	Orthotopic mouse (4T1.2)	Multifunctional polymeric nano micelles and intravenously injection	Inhibited metastasis formation and enhanced antitumoral immune response	Xu et al. (36)
	miR-36133-3p	miR-3613-3p mimic	MCF7, MDA-MB-231	SMS, PAFAH1B2, or PDK3	Xenograft mouse (MDA-MB-231)	Transfection and subcutaneously injection	Reduced the degree of pulmonary metastasis formation and primary tumor volume	Chen et al. (37)

### Antitumor miR-Based Therapy

In this paragraph, we discuss 16 *in vivo* studies that explored the ability of miRs to inhibit breast tumor growth, and the studies’ details are summarized in **Table 1**. Depending on the antitumor miR-based therapeutic strategy used, we grouped these studies into three subgroups: those focused on miRs replacement (seven studies), those focused on miRs inhibition (four studies), and those that used multiple miRs (one study).

Among the studies focused on the replacement of tumor-suppressor miRs, the older was published in 2012. Here, Li and colleagues designed an expression plasmid for miR-34a (T-VISA-miR-34a) to evaluate the antitumor effects of this miR as well as its mechanism of action *in vitro* ad *in vivo* experiments (13). The inducing of miR-34a expression suppressed BC cell growth, migration and invasion *in vitro* by downregulating the expression levels of the target genes E2F3, CD44, and SIRT1. Moreover, intravenous injection of liposomal nanoparticle formulation of T-VISA-miR-34a in an orthotopic mouse model of BC significantly led to tumor growth inhibition and prolonged survival without inducing systemic toxicity. Restoration of miR-34a in TNBC cell lines inhibited proliferation and invasion, activated senescence, and promoted cells sensitivity to dasatinib (14). Specifically, the authors found that miR-34a replacement significantly reduced c-SRC expression and affected the expression of many miR-34a targets embedded within the c-SRC signaling pathway including BCL2, NOTCH1, and IGF1R. Furthermore, miR-34a administration *in vivo*, significantly delayed tumor growth of orthotopically implanted tumors in mice and induced c-SRC downregulation. Again, to address enhanced antitumor therapy for TNBC, a combined therapeutic strategy of co-delivery of miR-34a and doxorubicin (DOX) was proposed throughout hyaluronic acid (HA)-chitosan (CS) nanoparticles (NPs) (15). The co-delivery of miR-34a and DOX achieved synergistic effects on tumor suppression, as it was able to enhance the antitumor activity of DOX by silencing Bcl-2 expression and suppressing BC cells migration *via* targeting Notch-1 signaling. Moreover, the nanocarrier-mediated co-delivery resulted in an effective reduction of drug resistance and side effects of DOX, improving its therapeutic outcome.

Other studies, focusing on miRs replacement have been published from 2016 to 2019 but very few of these studied the same miR. Wu et al. (11) found that miR-497 was highly down-regulated in BC tissue and immortalized BC cell lines and highlighted that its expression decreased under hypoxic conditions. Authors proposed miR-497 for BC therapy because they found that its administration, as miR-497-mimic, inhibited tumor growth and downregulated expression levels of VEGF and HIF-1α, suggesting its involvement in the inhibition of angiogenesis in a nude mouse xenograft cancer model. Zhu et al. (12) identified miR-544 as down-regulated in TNBC cell lines. They proposed this miR for the clinical treatment of TNBC because they found that increased miR-544 expression impaired migration, proliferation and invasion of TNBC not only in BC cell lines but also suppressed tumor growth *in vivo*, using a mouse xenograft tumor model. Bayraktar et al. (17) demonstrated that miR-603 expression in TNBC cell lines and tumors specimens was inversely correlated with eukaryotic elongation factor 2 kinase (eEF2K), which is a potential molecular driver in several cancers. They also showed that miR-603 expression impaired TNBC cell motility, migration and invasion by downregulation of eEF2K. Moreover, systemic administration of liposomal miR-603-NPs into TNBC xenograft mouse models led to significant inhibition of eEF2K expression and decreased activity of its downstream targets such as Src, Akt, cyclin D1 and c-myc, as a consequence the tumor growth was suppressed.

More recently, Yan et al. (18) developed nanosized liposomes functionalized with tLyp-1 peptide to specifically target the transmembrane glycoprotein receptor neuropilin (NPR) on BC tumor cells. This nanosystem loaded with miR-203 mimic determined post-transcriptional silencing of Slug and inhibition of the TGF-β1/Smad pathway *in vitro* and *in vivo*. Although these functional miR nanoliposomes were less effective at inducing apoptosis *in vitro*, the results showed their substantial ability to induce apoptosis in cancer-bearing mice and to exert a stronger anticancer efficacy and Slug silencing effect than functional vinorelbine liposomes. Moreover, the combined therapy with functional miR liposomes and functional vinorelbine liposomes resulted in nearly complete inhibition of tumor growth, leading to a remarkable anticancer effect in TNBC.

Despite targeting cancer cells only, miRs can also target the tumor-promoting stromal cells such as endothelial cells, tumor-associated fibroblasts or tumor-infiltrating immune cells which are involved in tumor formation and progression. Two examples are given by Tanno et al. (19) and Huang et al. (16), both aimed to reprogram tumor microenvironment (TME) through the targeted delivery of miRs. The former, after have identified *in silico* that miR-26a was significantly downregulated in TNBC tissues and associated with shorter overall survival, designed a miR aptamer as a platform to selectively deliver miR-26a to TNBC cells and hematopoietic stem/progenitor cells (HSPCs) (19). This platform suppressed tumor growth *in vitro* and enhanced the therapeutic effect of chemotherapy *in vivo*, protected hematopoiesis and significatively ameliorated myelosuppression associated with chemotherapy toxicity. The latter designed a multi-component system combining cationic *Bletilla striata* polysaccharide (cBSP), which contains high mannose moieties, with PEG-histamine-modified alginate (PHA), a pH-responsive material, for targeting the delivery of synthetic mir-let-7b mimic (16). In this way, was obtained a miR-delivery complex with a high affinity for the mannose receptors on tumor macrophages (TAMs) and tumor-infiltrating dendritic cells (TIDCs) that could specifically release let-7b in response to the TME. *In vivo* administration of let-7b, efficiently reprogrammed the functions of TAMs/TIDCs, *via* stimulating TLR-7 signaling and suppressing the IL-10 production, reversed the suppressive TME, inhibited tumor growth and increased survival of BC mouse model. Six studies, between 2013 and 2019 years, focused on the inhibition of oncomiRs, and five of them recognizing miR-21 as a potential miR for BC treatment. In (21), the authors found that antagomiR-21 treatment suppressed proliferation and induced apoptosis *via* targeting PTEN in murine 4T1 cells. Moreover, they found that miR-21 inhibition strongly retarded breast tumor growth and inhibited angiogenesis by suppressing the HIF-1a/VEGF/VEGFR2 pathway *in vivo* experiments. More recently, based on a stable core scaffold of the pRNA-3WJ motif previously realized (38), Yin et al. designed multifunctional RNA NPs to deliver antagomiR-21 (22). To improve targeting specificity, the branch of pRNA-3WJ was modified with an RNA aptamer against CD133 (CD133apt) that is a cell surface marker used to characterize cancer stem cells highly expressed in TNBC patients. Negatively charged, small size and CD133 aptamer conferred to RNA NPs high tumor-specific targeting and low immunogenicity. *In vitro* and *in vivo* experiments demonstrated that miR-21 knockdown upregulated the tumor suppressors PTEN and PDCD4. Moreover, in the orthotropic TNBC mouse model, the treatment with pRNA-3WJ/CD133apt/antagomiR-21 resulted in favorable tumor targeting and effective tumor growth inhibition. We found also two examples of co-delivery of antagomiR-21 together with chemotherapeutic compounds to enhance their efficacy in BC treatment. In the former, antagomiR-21 and DOX were encapsulated in PAMAM modified gold NPs (23). This delivery system was tailored to achieve programmed delivery driven by NIR of antagomiR-21 first and DOX then. This sequential delivery produced a huge synergistic apoptotic response *in vitro* and reduced the stemness, as indicated by the reduction of marker Nanog, Oct4, and Sox2. Moreover, sequential release therapy resulted also in an effective inhibition in tumor growth *in vivo*. In the latter study, the co-delivery of antimir-21 and epirubicin (Epi) was performed throughout a targeted delivery system made of two biocompatible polymers of (poly β-amino-ester and poly d, l-lactide-*co*-glycolide), and modified with MUC1 (24). The co-delivery of antagomiR-21 and Epi led to escalating cytotoxicity for target cancer cells and remarkably inhibited tumor growth in tumor-bearing mice compared with chemotherapic drug alone.

Otherwise, Devulipally et al. focused on antagonize multiple endogenous miRs, co-loading antisense-miR-21 and antisense-miR-10b in polymeric NPs (made of block copolymer Polylactic-*co*-glycolic acid and Polyethylene glycol, PLGA-b-PEG NPs) modified with urokinase plasminogen activator peptide (uPA) for specific targeting of TNBC (25). Authors found that antagonizing multiple miR activities had a cumulative effect in reducing BC cell proliferation both *in vitro* and *in vivo*. Indeed, the sustained release of antisense-miRs over 15 days achieved a 40% reduction in tumor growth compared to the control in tumor xenografts. At last, to decrease BC cell proliferation *in vitro* and *in vivo*, Naseri and colleagues (20) assessed whether MSCs mesenchymal stem cells (MSCs)-derived exosomes could act as a carrier to deliver LNA (locked nucleic acid)-modified miR-142-3p inhibitor to murine BC tumor cell line (4T1 and TUBO). They found that exosome-mediated delivery of miR-142-3p inhibitor reduced tumorigenicity of BC *in vitro* and *in vivo*.

Finally, Conde et al. (26) exploited a dual miR therapy with simultaneous inhibition of oncogenic miR-221 and replacement of tumor suppressor miR-205. An RNA-triple-helix hydrogel scaffold loaded with the RNA oligonucleotides was realized for local anticancer therapy and implanted adjacent to the tumor in the mammary. This system afforded to be highly efficacious to downregulate miR-221 with following the increase of E-cadherin levels and decrease of Snail and Slug expression levels, to enhance the immunosuppression capacity, resulting in potent and long-lasting tumor reduction of nearly 90%. Moreover, mice implanted with RNA-triple-helix hydrogel scaffolds showed a highly significant survival advantage.

### Antimetastatic miR-Based Therapy

Prediction and treatment of metastasis are critical for enhancing the survival of BC patients, as metastatic BC remains a disease with a poor prognosis and about 30% of women diagnosed with an early stage will have a secondary progression (39). In this paragraph, we review *in vivo* studies that explored the antimetastatic power of miRs for BC treatment. In total, nine articles are discussed here, and the studies’ details are summarized in **Table 2**. According to the strategy used for their classification, we found that six studies focused on miRs inhibition and three on miRs replacement. As early as 2010, Ma et al. focused on the pharmacological inhibition of a master regulator of metastatic cell viability, the miR-10b (27). This study demonstrated that the therapeutic silencing of miR-10b inhibited metastasis in a mouse mammary tumor model. In particular, administration of miR-10b antagomiRs to mice bearing highly metastatic cells (4T1) did not reduce primary mammary tumor growth but suppressed the formation of lung metastases. To determine whether antagomiR-10b had some effect on tumor cells that had already disseminated, authors used as a route of transplantation tail vein injection of 4T1 cells by introducing cancer cells directly into the lung microvasculature, thus avoiding the initial steps of the invasion-metastasis cascade. In this setting, both the control group and antagomiR group of mice developed similar numbers of lung metastases, suggesting that antagomiR-10b did not affect the late stage of the metastatic process. Working on the same target, another more recent study focused on a very aggressive model of stage IV metastatic BC and realized a miR-10b inhibitory nano-drug system, made of magnetic NPs (MN-NPs) conjugated to LNA-based miR-10b antagomiRs (28). Intravenous injection of this nano-drug into mice model of metastatic BC (4T1 orthotopic model) resulted in selective targeting and elimination of metastatic tumor cells (28). Moreover, weekly treatment with the nano-drug in combination with a low dose of DOX resulted in complete regression of pre-existing distant metastases in 65% of the animals and in a significant reduction of cancer mortality compared to control groups, including a group treated exclusively with a standard dose of DOX. Zhang and colleagues too focused on the pharmacological inhibition of miR-10b, but they performed the co-delivery of antagomir-10b with paclitaxel (PTX) by a liposomal delivery system modified with a pH-responsive anti-microbial peptide [D]-H_6_L_9_ (D-Lip). This approach significantly hindered the migration of 4T1 cells and induced cellular apoptosis *in vitro* (29). Moreover, the combined delivery of antagomir-10b and PTX delayed the growth of 4T1 tumors, reduced the lung metastases, and significantly up-regulated the expression of Hoxd10 in tumor site.

Dettori et al. have explored the link between miR-214 overexpression and metastasis formation. This miR was found up-regulated in TNBC cell lines and tissues and promotes metastatic dissemination. In their experiments, the authors used two antagomiR-214 compounds, R97 and R98 (antagomiR-214 antagomiR-214 compounds contains a full phosphorothioate backbone and sugar modifications, at diverse positions, along the nucleotide sequence) and tested their antimetastatic therapeutic silencing (30). When 4175-TGL BC cells were transfected with R97/R98, miR-214 expression reduced and transendothelial migration, responsible for their metastatic traits, resulted impaired. Noteworthy, when mice bearing primary BC tumor (4175-TLG miR-214over) were treated with R98 antagomiR-214 (every other day until for 25 days), the mass measurement of the primary tumor resulted statistically unchanged, while metastatic dissemination from the primary tumor to lung resulted in strongly reduced between R98-treated and control mice. Interestingly, the authors found that when antagomiR-214 compounds were systemically delivered to mice carrying BC or melanoma, a reduced number of circulating tumor cells (CTCs) and lung or lymph node metastasis formation resulted.

In the wake of oncomiRs silencing, several studies apply miR-based therapeutic approach to modulate TME for impairing metastasis formation. A study that goes in this direction was performed by Bliss and colleagues, that aimed to arrest BC recurrence targeting the dormant BC cells in bone marrow (31). After investigating the mechanisms by which MSCs communicate with BC cells through exosomes to impart cycling quiescence, authors developed MSC-loaded with antagomiR-222/223 in combination with a reduced dose of carboplatin, thus managing to chemosensitize and eradicate BC cells in an *in vivo* model of BC dormancy (Oct4^hi^ sorted MDA-MB-231 or T47D xenograft mouse). To analyze the involvement of the TME in the metastasis development, De Cola and colleagues investigated the role of miR-205 in BC progression and showed that miR-205-5p silencing impaired the metastatic potential of BC stem cells *in vitro* and *in vivo* modulating endothelial–mesenchymal transition (EMT) process (32). Using specific LNA-oligonucleotides, the silencing of miR-205-5p impaired tumor growth and reduced the number and the size of lung metastasis *in vivo*. Also, Wang et al. (33) after evaluating the upregulated expression of miR-100 in cells and BC tissues, assessed that miR-100 promoted the M2-polarization of macrophages and maintained tumor-associated macrophages (TAMs) phenotype both *in vitro* and *in vivo*, *via* mTOR signaling pathway regulation and upregulation of IL-1ra secretion. The inhibition of miR-100 significantly impaired lung metastasis and increased the chemosensitivity of tumor cells to cisplatin.

The last three studies focused on BC metastasis suppression by tumor suppressor miRs replacement. Zhao et al. investigated the role of miR-4306, disclosing that it was epigenetically regulated by the loss of ER-α, HER2 and PR (34). Upregulation of miR-4306 suppressed TNBC metastasis, particularly lymph node metastasis, and inhibited angiogenesis and lymphangiogenesis, predominately through direct downstream targeting of three molecules such as vascular endothelial growth factor A (VEGFA), Homeobox protein SIX1 and cell division control protein 42 (Cdc42), yet reported as oncogenes in BC. Administration of a cholesterol-conjugated miR-4306 mimic in combination with cisplatin in an orthotopic mouse model of TNBC, inhibited tumor growth and suppressed metastasis. Another recent example of miR-replacement therapy using nanocarriers is the study of Ramchandani et al. (35). The study proposed the miR-708 as a therapeutic agent able to make the malignant progression of breast neoplasia less aggressive and more manageable by standard regimens. Authors realized Gold NPs (AuNPs) coated with alternate multilayers of poly-L-lysine (PLL) and miR-mimic. This multilayered assembly made possible the sustained release of miR-mimic *in vitro* as well as *in vivo*. The administration of these AuNPs in the orthotopic BC tumor model (MDA-MD-231-LM2) increased miR-708 expression of the primary tumor in mice and significantly impaired lung metastases formation compared with group control. Moreover, the authors discovered that miR-708-NP targets a unique OCT4/SOX2þ miR-708 low subpopulation of cancer cells featured by self-renewing and highly migratory phenotype. In the same way, Xu et al. (36) developed a multifunctional polymeric nanomicelle based on the conjugation of amphiphilic copolymer POEG-VBC backbone with creatine for systemic co-delivery of a bioengineered miR prodrug (tRNA-miR-34a) and DOX. This therapeutic approach exhibited a significant synergistic antitumor and antimetastasis activity both *in vitro* and *in vivo* because it induced apoptosis, necrosis and enhanced immune cell infiltration to the tumor-bearing site, contributing to the overall antitumor efficacy without inducing much cytotoxic effect.

Recently, a study has demonstrated the deletion of MIR3613 in BC genome, its correlation with the molecular subtypes, and with an unfavorable prognosis in estrogen receptor-positive BC patients (37). Furthermore, miR-3613-3p levels resulted decreased in serum or BC tissues of affected patients. *In vitro* experiments proved that miR-3613-3p suppressed proliferation and sphere formation, promoted apoptosis and could restrain tumor progression because affecting on cell cycling pathway by targeting SMS, PAFAH1B2, or PDK3. Additionally, administration of miR-3613-3p in an immunocompromised mouse model of human BC cell MDA-MB-231 significantly reduced volumes of tumor and the degree of pulmonary metastasis formation.

## Discussion

miRs deregulation is associated with alteration of downstream gene networks and is a common feature underpinning different disease. Therefore, in the last decades, many research areas have focused on disease modification by restoring homeostasis of dysregulated processes through the development of miR therapeutics. miR-based therapeutics appear particularly suited for the treatment of an extensive range of human conditions, above all for complex and multigenic disorders, including cancer disease (40). The global miR market size was valued at $ 160.5 million in 2017 and is expected to grow by 18.6% during 2018–2025 (41). Currently, many academic laboratories, pharmaceutical industries and biotech companies are involved in miR therapeutics development and numerous candidate miR therapeutics are in clinical development, in phase I and II clinical trials (42). The first miR that is rapidly moving from bench to clinic is LNA-antagomiR-122, named miravirsen, for the treatment of hepatitis C virus (HCV) infection (43). It is a modified oligonucleotide, made up of 15 nucleotides, that binds and inhibits miR-122. It is now in phase II clinical trial (NCT01200420) undergoing assessment for its safety and effectiveness in the patients. To date, just a few miR-based therapeutics are actually under Phase-I clinical trials for oncological diseases (see **Table 3**), such as Cobomarsen and TargomiR, both developed by miRagen Therapeutics. Cobomarsen, (also known as MRG-106) is an inhibitor of miR-155, an oncomir highly expressed in a wide range of cancers, including BC (44). Two clinical trials are ongoing in Phase I, to evaluate the safety of this drug for the treatment of adult patients diagnosed with certain lymphomas and leukemias (NCT02580552 and NCT03713320). TargomiR consists of targeted minicells containing a miR-16 mimic. This drug has completed Phase I recruitment (NCT02369198) of patients with malignant pleural mesothelioma and non-small-cell lung cancer and the first published report showed partial response and no adverse effects in the entire patient cohort (45). Furthermore, a special amphoteric lipid nanoparticle filled with miR-34 mimics, named MRX34, was tested in a clinical trial (NCT01829971). This trial recruited 155 participants, seven cancer types in all, including primary liver cancer, several solid tumors and hematopoietic malignancies. Although Phase I trial of this drug was halted, due to severe immune-related adverse events experienced in five patients, this clinical trial provides a direction for MRX34 application on oncotherapy and above all the proof-of-concept for miRNA based cancer therapy. In support of this, MRX34 treatment with dexamethasone premedication resulted in a manageable toxicity profile in most treated patients (46, 47), revealing the need to anticipate toxic effects from this class of drugs, specifically immune-mediated events, that might not always be seen in pre-clinical models. To support of this, Zhang et al. have recently discussed the delayed development of miRNA in a pharmacological perspective, arguing that the response to the occurrence of adverse events, and thus the disrupted miR-therapeutic clinical trial pipeline, is a consequence of “too many targets for miRNA effect” (48). In general, a miRNA targets tens to hundreds of genes; this means that miRNA therapies could trigger unknown and non-preventable consequences, just as immune-related events.

**Table 3:** Clinical trials of miR-therapeutics in human cancers.

Drug Name (Company)	Therapeutic miR	Clinical trials gov. Identifier	Current Trial status	Cancer type
MesomiR-1 (TargomiRs) (EnGeneIC)	miR-16 mimic	NCT02369198	Phase I (completed expected to enter phase II)	Malignant pleural mesothelioma Non-small cell lung cancer
MRX34 (Mirna Therapeutics)	miR-34 mimic	NCT01829971	Phase I (terminated due to immune-related toxicities and deaths)	Primary Liver Cancer Small cell lung cancer Lymphoma Melanoma Multiple Myeloma Renal Cell Carcinoma Non-small cell lung cancer
MRG-106 (miRagen Therapeutics)	AntagomiR-155	NCT02580552	Phase I (recruitment completed)	Cutaneous T-cell Lymphoma Mycosis Fungoides Chronic Lymphocytic Leukemia Diffuse Large B-Cell Lymphoma ABC Subtype Adult T-Cell Leukemia/Lymphoma
MRG-106 (miRagen Therapeutics)	AntagomiR-155	NCT03713320	Phase II (recruitment completed)	Cutaneous T-Cell Lymphoma/Mycosis Fungoides

Despite the huge number of patents granted for “miR and Breast Cancer” (41), there is no RNA-based drug for BC treatment that has yet reached the clinical trial pipeline. Here, we systematically analyzed the potential of miR-based therapeutics to achieve BC treatment. We have carried out a systematic review of all studies in which miRs were assessed *in vivo* as therapeutics or as therapeutic targets to be silenced. In this qualitative analysis, starting from 174 identified records, we selected a total of 27 studies that met the inclusion criteria we set for writing this systematic review. Due to their high heterogeneity, we grouped the 27 eligible studies according to the main therapeutic skills of miRs studied: antitumoral and antimetastatic miR-based therapy. Overall, on 27 reviewed studies, we found 19 different miRs tested as potential therapeutics or potential therapeutic targets in animal model experiments. About 90% of the selected studies developed mouse models of TNBC to evaluate the efficiency and the safety of miRs as therapeutic molecules. This data confirms how much research forces are projected towards this tumor subtype, which remains the most lethal form of BC and the biggest challenge to face in BC treatment.

Among all systematically reviewed miR-based therapeutics, miR-10b, miR-21, and miR-34a emerged to be those most investigated. MiR-21 plays an oncogenic role in BC (49) and four studies (21–23, 25) proposed it as a potential antitumoral therapeutic for TNBC treatment. These examples of miR- inhibition therapeutic approaches agreed on obtained inhibited tumor growth by regulating signaling pathways involved in apoptosis and tumor suppressor genes including PTEN, PDCD4 and VEFG (21, 22), and also in stemness (23). Moreover, the synergic delivery of antimir-21 with chemotherapic drug (Epi) showed significantly enhanced antitumor efficacy (24). Despite these encouraging results, it is worth highlight that miR-21 is a common key regulator of cellular mechanisms such as apoptosis, proliferation and migration and lacks specificity because it is deregulated not only in BC but also in many other cancers and other human pathologies (50). MiR-10b is known to be a metastasis promoting factor across many cancer types (51). It was shown that at the receiving end of the miR-10b pathway is the proto-oncogene c-Jun (52), a transcription factor that plays a critical role in the stimulation of cell proliferation and tumor progression. Here, we found successful examples of how inhibition therapy throughout antagomir-10b could suppress the formation of distant metastasis (27–29), and also delayed the growth of the primary tumor (29). Also miR-34a emerged from our systematic review of literature as one of the most assessed miR-therapeutic for BC. MiR-34a is a tumor suppressor (53), it downregulates the expression of many oncogenes across multiple oncogenic pathways, of genes involved in tumor immune evasion, and it is lost or under-expressed in many malignancies (46). Its therapeutic replacement *in vivo* BC models exhibited antitumor effects (13, 15), enhanced antitumoral immune response (36, 54), and also suppressed BC cell migration (15) and promoted antimetastatic activity (36, 54). Furthermore, miR-34a was also involved in sensitizing BC to traditional chemotherapeutic agents (15). These promising results can be translated well to other epithelial carcinomas (55), as miR-34a inhibited the growth of primary tumors, blocked metastasis and extended survival in various preclinical animal solid tumor models different from BC, as prostate (56), liver (57) and lung cancer (58). Besides, another recurrent miR that emerged from our systematic analysis is miR-205, that showed a double miR-therapeutic power with antitumoral and antimetastatic effects: its tumor-suppressive activity was exploited by a miR-replacement approach (26), while its oncogenic role was targeted using a miR-inhibition approach (32). These results are linked to dual roles that miRs can have since they can target both oncogenes and tumor suppressors. Therefore, as well as there are miRs that exert similar functions in different tumor types, it is not rare that miRs could have specific context-dependent roles. These are very important issues that need to be carefully considered for driving an appropriate selection of miRs for therapeutic purposes and develop reliable and safe miR-based therapy.

In our search, we came across studies focusing on miRs that act on components of the TME such as fibroblasts, endothelial cells, and immune cells. Indeed, it is well known that miRs are used by cancer cells to alter and shape a protumorigenic milieu by a bidirectional interaction with surrounding cells (59). So, here we overview encouraging evidence about the using of miR-based therapeutics to modulate TME and to impair BC tumor growth (16, 19, 20) and metastasis formation (31–33). Indeed, miR-based therapeutics that target TME components demonstrated to be useful approaches to reverse TME suppressive function and enhance sensitivity to chemotherapy drugs (19, 33).

To allow the development of miR-based therapeutics, main challenges such as stability and delivery must be addressed (60). *In vivo* delivery of antagomiRs/miR mimics as cancer therapies should include the effectiveness and the accurateness of these molecules to the target cells, that instead encounter several barriers such as blood clearance, enzyme degradation, and intracellular trapping, and poor bioavailability. For systemic miR delivery for cancer treatment, two are the types of used vectors: viral and nonviral carriers. Although nonviral carriers display generally less efficient miR delivery and short efficacy compared to viral carriers, they are considered the preferred choice because of less immunogenicity (61). Here, the most recurrent approaches developed to ensure an efficient *in vivo* delivery of miRs to target BC included nanocarriers, some as liposomal NPs (13, 14, 17, 18), others as polymeric NPs (24, 25, 36), hydrogel NPs (15, 16, 26), and other as metallic NPs (22, 28, 35). Moreover, a common result of co-administration of standard treatments with miR-based therapeutics through nanocarrier formulation resulted in enhance drug bioavailability and improve the antitumoral and antimetastatic effect.

The major limitation of this systematic review was the lack, in our query results, of studies focused on miRs able to regulate immune checkpoints for immunotherapy purposes in BC. So, we felt it could be useful to argue this new field of study in the discussion section. In the last years immunotherapy has emerged as an advanced and powerful aids promising to treat various cancers (62, 63). The anti-PD-L1 monoclonal antibody Atezolizumab has received FDA approval in March 2019, since the IMpassion130 trial showed a significant increase in overall survival (OS) for patients with PD-L1 positivity (PD-L1 IC ≥1%), treated with Atezolizumab plus nab-paclitaxel versus nab-paclitaxel alone as first-line therapy for metastatic TNBC. Additionally, VENTANA PD-L1 (SP142) assay, to determine PD-L1 positivity, was FDA approved by results from the Impassion130 study (64, 65). Again, pembrolizumab has demonstrated promising results in early-stage TNBC and could be near its approval in the (neo) adjuvant setting (66). In addition to approved checkpoint inhibitors able to blockade more checkpoint proteins (CTLA-4, PD-1 and PD-L1) acting as breakers on the active immune system (67), increasing evidence suggests that miRs and immune checkpoint molecules are tightly interrelated so that specific miRs might have direct clinical relevance (68). Among the immune checkpoint molecules, the miR-based regulation of PD-L1 is the most investigated, as many tumors, including BC, overexpress PD-L1 on cellular surfaces, which inhibits the proliferation of T cells by binding to its receptor PD-1 (69). Many studies introduced several tumor-suppressor miRNAs targeting PD-L1 in different human cancers including miRNA-16, miRNA-15b, miRNA-15a, miRNA-193a-3p (70), miRNA-106b-5p and miRNA-93-5p (71), miRNA-142-*5p (72), miRNA-152 (73), miRNA-324-5p or miRNA-338-5p (74), and miRNA-138-5p (75), miR-200 (76), and miR-34a (77). But, in the last two years, the number of studies focusing on miRs able to target PD-L1 in BC, especially in the TNBC subtype, is growing up rapidly. These studies explore the potential use of miRs as immune checkpoint inhibitor in BC. MiR-138-5p (78), miR-383-5p (79), miR-424-5p (80), miR-200 (81), miR-148a-3p (82), miR-3609 (83) and miR-873 (84) have been all identified as potentially able to bind and downregulate the expression of PD-L1 in BC cell lines and in some of them *in vivo* too. All these miRs have been found deregulated in BC cell lines, where the PD-L1 was instead overexpressed, and luciferase assays demonstrated that these miRs could bind the 3’UTR of PD-L1 mRNA. Ectopic expression of these miRs inhibited cell proliferation and motility as well as induced apoptosis. Interestingly, in (79) was evaluated the effect of miR-383-5p on T-cells co-cultured with transfected BC cells and authors found that miR-383-5p increased the expression of INF-γ, TNF-α, and IL-2 and decreased the expression of TGF-β and IL-10, highlighting the focal role of miR-383-5p in suppressing tumor development and inducing pro-inflammatory tumor microenvironment in BC.

Taken together, the broad involvement of miRNAs in modulating immune checkpoints as well as tumor microenvironment give miRNAs further promising power as immune therapeutic agents.

## Conclusion

In conclusion, due to the complexity and heterogeneity of BC, the use of miR-based therapeutics for BC treatment seems appealing because, being pleiotropic molecules, they can modulate multiple dysregulated genes and/or pathways simultaneously. Nevertheless, their pleiotropic role can represent a double-edged sword because, until all the possible molecular targets of each miR will not be fully known, their inhibition or activation for therapeutic purposes will not be completely manageable from a clinical point of view. Despite their power and although multiple companies are investing significant efforts to develop miR-based drugs, miR-based therapeutics seems to be an immature field that has not so reached yet the clinical application. Indeed, none of the selected studies in this review goes beyond preclinical studies. Therefore, the main hurdle for the future development of this class of therapeutic molecules is to expand the studies related to miRs according to a deeper knowledge of their targets to provide a more comprehensive overview of molecular pathways that they can modulate. Thus, we think this knowledge could drive miR-based therapeutics beyond Phase II.

## Data Availability Statement

The original contributions presented in the study are included in the article/**Supplementary Material**. Further inquiries can be directed to the corresponding author.

## Author Contributions

MI and AMG contributed to conception and design of the study. AMG and MI collected and organized data. AMG wrote the first draft of the manuscript. MI and MS reviewed and edited the manuscript. All authors contributed to the article and approved the submitted version.

## Funding

This research was funded by the Ministry of Health under contract “Ricerca Corrente RRC-2020-23669967” to MI.

## Conflict of Interest

The authors declare that the research was conducted in the absence of any commercial or financial relationships that could be construed as a potential conflict of interest.
